# Validation of simple measures of aortic distensibility based on standard 4-chamber cine CMR: a new approach for clinical studies

**DOI:** 10.1007/s00392-019-01525-8

**Published:** 2019-07-13

**Authors:** Lukas Stoiber, Niky Ghorbani, Marcus Kelm, Titus Kuehne, Nina Rank, Tomas Lapinskas, Christian Stehning, Burkert Pieske, Volkmar Falk, Rolf Gebker, Sebastian Kelle

**Affiliations:** 1 Department of Internal Medicine/Cardiology, German Heart Center Berlin, Berlin, Germany; 2 Department of Cardiothoracic and Vascular Surgery, German Heart Center Berlin, Berlin, Germany; 3 Department of Congenital Heart Disease/Pediatric Cardiology, German Heart Center Berlin, Berlin, Germany; 4 Department of Cardiology, Medical Academy, Lithuanian University of Health Sciences, Kaunas, Lithuania; 5 Department of Internal Medicine and Cardiology, Charité-Universitätsmedizin Berlin, Berlin, Germany; 6 DZHK (German Centre for Cardiovascular Research), Partner Site, Berlin, Germany; 7 Philips Health Care, Hamburg, Germany

**Keywords:** Aortic distensibility, Compliance, Cardiac magnetic resonance imaging, Cine MRI, Reproducibility

## Abstract

**Objective:**

Aortic distensibility (AD) represents a well-established parameter of aortic stiffness. It remains unclear, however, whether AD can be obtained with high reproducibility in standard 4-chamber cine CMR images of the descending aorta. This study investigated the intra- and inter-observer agreement of AD based on different angles of the aorta and provided a sample size calculation of AD for future trials.

**Methods:**

Thirty-one patients underwent CMR. Angulation of the descending aorta was performed to obtain strictly transversal and orthogonal cross-sectional aortic areas. AD was obtained both area and diameter based.

**Results:**

For area-based values, inter-observer agreement was highest for 4-chamber AD (ICC 0.97; 95% CI 0.93–99), followed by orthogonal AD (ICC 0.96; 95% CI 0.91–98) and transversal AD (ICC 0.93; 95% CI 0.80–97). For diameter-based values, agreement was also highest for 4-chamber AD (ICC 0.97; 95% CI 0.94–99), followed by orthogonal AD (ICC 0.96; 95% CI 0.92–98) and transversal AD (ICC 0.91; 95% CI 0.77–96). Bland–Altman plots confirmed a small variation among observers. Sample size calculation showed a sample size of 12 patients to detect a change in 4-chamber AD of 1 × 10^−3^ mmHg^−1^ with either the area or diameter approach.

**Conclusion:**

AD measurements are highly reproducible and allow an accurate and rapid assessment of arterial compliance from standard 4-chamber cine CMR.

**Graphic abstract:**

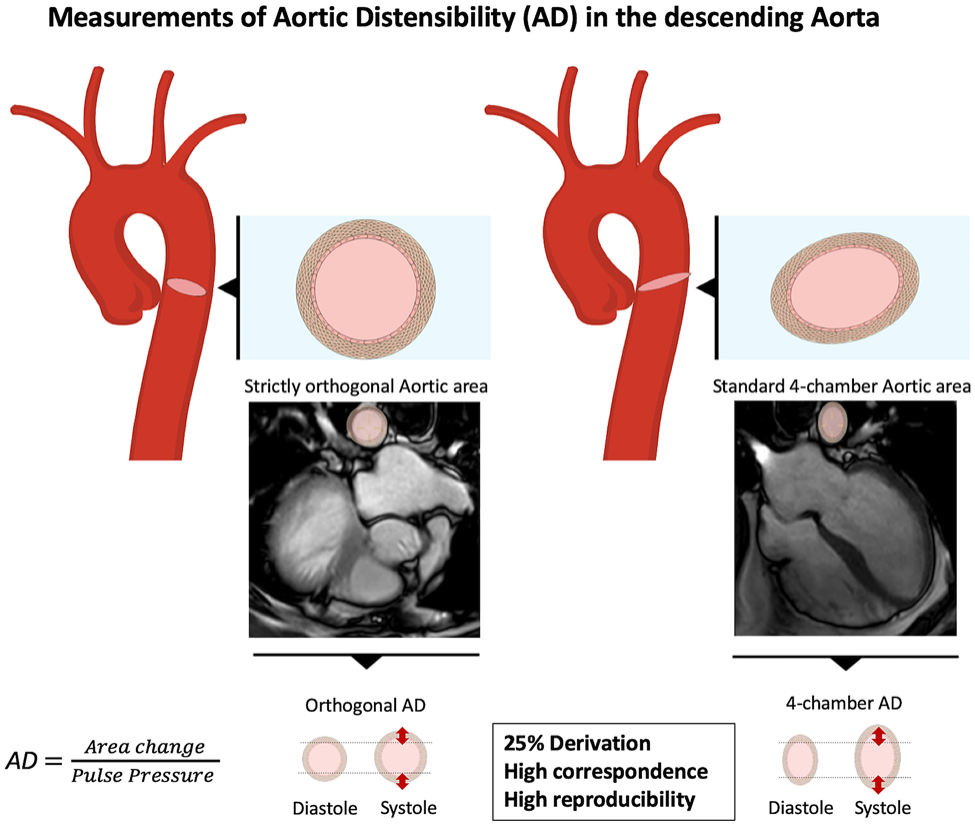

**Electronic supplementary material:**

The online version of this article (10.1007/s00392-019-01525-8) contains supplementary material, which is available to authorized users.

## Introduction

Arterial compliance, the tendency of blood vessels to stretch in response to the pulsatile blood flow, has significant physiological effects on blood pressure (BP) [1–3]. The cushioning function of the aorta may be impaired due to structural degeneration of the aortic wall, increasing stiffness and thus the afterload for the left ventricle [3].

Aortic distensibility (AD) provides an estimation of these elastic properties and normalizes arterial compliance with the size of the vessel, allowing for a better comparison between individuals [4].

Previously, several authors have made significant contributions to assess aortic compliance with various imaging methods [5–8]. Measurements of AD can give information even on subclinical vascular changes and are being investigated as predictors of cardiovascular morbidity [4, 9–11]. Newer preclinical and clinical studies are focusing on mechanisms of influencing AD. Moreover, we recently showed AD to be a potential biomarker to act as a non-invasive control in interventional hypertension trials [4].

Currently, cardiac magnetic resonance tomography (CMR) is regarded as a gold standard to assess areas of different parts of the aorta to calculate AD. However, some aspects regarding the meaningfulness and interpretability of AD in the descending aorta remain unresolved. Even though the descending aorta is incidentally imaged in every standard 4-chamber view cine steady-state free precession sequence (SSFP) of the heart, it remains uncertain whether these images can be used to calculate AD in daily clinical practice adequately. The current study aimed to systematically analyze reproducibility (with intra- and inter-observer agreement) of cine imaging in the evaluation of AD. Our measurements were acquired using various types of angulation of the descending aorta in CMR.

## Methods

**Fig. 1:**
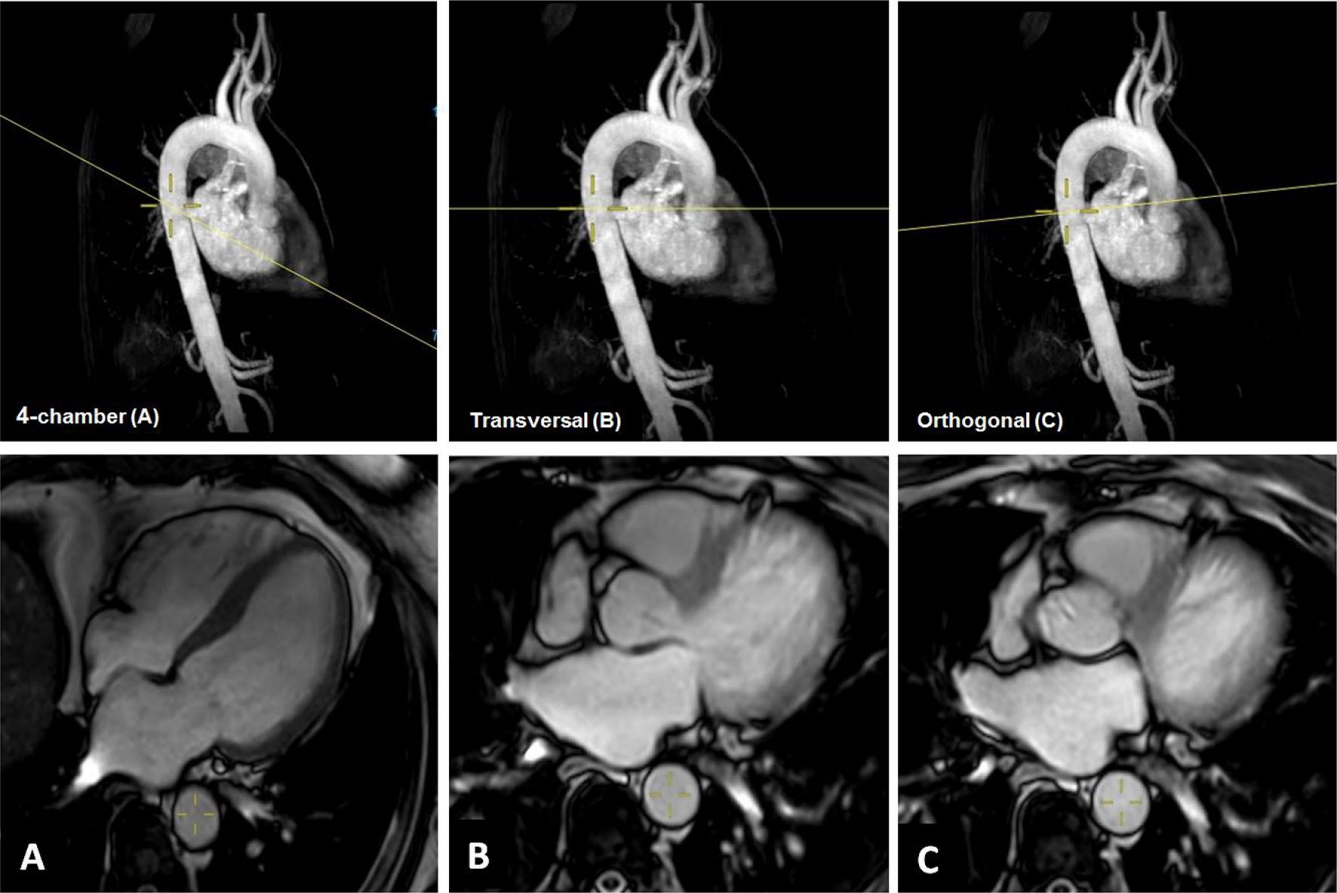
Illustration of CMR angulation of the descending aorta at the time of image acquisition and corresponding 4-chamber (**a**), transversal (**b**) and orthogonal (**c**) aortic areas. Image **a** shows a standard 4-chamber SSFP image where the slightly oval areas of the descending aorta can easily be tracked without further technical planning. Images **b** and **c** demand proper planning and are not performed in daily practice clinical imaging of the heart

We performed CMR in 31 subjects (mean age 57 years) with variable indications. The study complies with the Declaration of Helsinki. Institutional Review Board approval was not necessary due to a retrospective analysis of clinical data. According to local law, all individuals signed informed consent before entering the clinical MRI. The data were anonymized and none of the observers had the possibility of identifying patient information when analyzing the data. All images were acquired using a Philips Ingenia 3.0 Tesla Scanner (Philips Healthcare, Best, the Netherlands). Cine images were acquired during breath holds of 10–15 s using vector electrocardiogram gating and steady-state free precession sequence [4]. All study participants were scanned using the same imaging protocol, which consisted of angulation of the descending aorta to obtain strictly transversally and orthogonally cut cross-sectional areas of the aorta [16]. Fifty images per cardiac cycle were obtained. AD was determined as the change in the cross-sectional aortic area per unit change in BP, as previously reported [4, 10, 16, 17]. Office BP was obtained during the MRI with an automatic brachial oscillometric monitor after at least 5 min of rest [4]. Two experienced observers then performed post-processing of the CMR dataset with the Medis Suite Version 2.1. (Medis, Leiden, The Netherlands). Both observers had more than 2 years of experience with general CMR imaging. Qmass software was used to contour the inner diameter of the aortic wall. Maximum and minimum aortic areas were calculated by (i) tracing the largest and smallest extension of the aortic wall contour throughout the cardiac cycle and (ii) tracing aortic diameters to calculate a hypothetic circle aiming to obtain a strictly circular aortic area. We first assessed cross-sectional areas of the descending aorta obtained in standard 4-chamber cine images. In a second step, we performed the same measurements in images based on strictly transversal or orthogonal cuts. Figure 1 illustrates the three different angles used at the time of image acquisition. All measurements were repeated three times and then averaged. To calculate AD, we first determined aortic strain, defined as the relative change in area, and then normalized this value with the peripheral pulse pressure (PP) obtained at the time point of the CMR (average of three PP values). The formula used for the AD calculation has been published previously [4, 10, 16, 17].

### Sample size calculation

To detect a clinically significant change of 0.5, 0.8 and 1 × 10^−3^ mmHg^−1^ in aortic distensibility with the power of 90% and a significance level (*α*) of 5%, the sample size calculations were performed using the following formula:

$$n = f \left( { \propto ,P} \right) \cdot \sigma^{2} \cdot 2/\delta^{2}$$

where *α* is the significance level, *P* the required power, *n* the sample size and *f* the value of the factor for different values of *α* and *P* ($f$ = 10.5 for $\propto$ = 0.05 and *P* = 0.090), with $\sigma$ the standard deviation of differences between two measurements and $\delta$ the target difference to be detected [18, 19].

### Statistical analysis

All data are presented as mean ± standard deviation. Differences in mean values were compared using Student’s *T* test if data were normally distributed or the Wilcoxon test if normality could not be assumed. Kolmogorov–Smirnov test was used to assess distribution. Univariate correlations between parameters were obtained using Pearson’s correlation coefficients. Intra- and inter-observer variability is displayed in Bland–Altman plots. The intra-class correlation coefficient (ICC) was considered excellent with a value of > 0.7 [20]. A *P* value < 0.05 was considered statistically significant. Statistical analysis was performed using IBM SPSS Statistics for Windows (Version 24.0, SPSS Inc., Chicago, IL, USA).

## Results

### Study population

Thirty-three patients were included in this analysis. Two patients had to be excluded due to low image quality. The mean age was 57 years, 13/33 (39%) were female and mean BP was 122/68 mmHg.

### Values of aortic areas, aortic strain, and aortic distensibility

Table 1 provides an overview of the minimal and maximal aortic areas, aortic distensibility and strain derived from either aortic area or aortic diameter. The values displayed are those obtained from observer 1. Pearson correlation coefficients are represented.

**Table 1:** Overview of minimal and maximal aortic areas and aortic distensibility and strain derived from either aortic area or aortic diameter. Pearson correlation coefficients are represented. Values represent measurements of observer 1

Parameter (*n* = 31)	Area	Diameter	Pearson CC	*P* value CC*
Minimal areas of descending aorta (mm^2^)				
Transversal angulation	445.09 ± 178.65	419.11 ± 156.24	0.993	< 0.001
Orthogonal angulation	442.35 ± 159.38	420.86 ± 146.24	0.994	< 0.001
Classic 4-chamber angulation	482.82 ± 167.14	432.24 ± 153.93	0.986	< 0.001
Maximal areas of descending aorta (mm^2^)				
Transversal angulation	497.67 ± 184.50	476.04 ± 161.41	0.989	< 0.001
Orthogonal angulation	487.51 ± 159.62	470.08 ± 149.95	0.992	< 0.001
Classic 4-chamber angulation	549.18 ± 171.16	487.60 ± 156.93	0.971	< 0.001
Aortic strain (mm^2^)				
Transversal angulation	13.08 ± 6.44	14.76 ± 6.35	0.819	< 0.001
Orthogonal angulation	11.67 ± 6.77	13.00 ± 6.97	0.785	< 0.001
Classic 4-chamber angulation	15.27 ± 7.38	14.42 ± 7.71	0.798	< 0.001
Aortic distensibility (10^−3^ mmHg^−1^)				
Transversal angulation	2.80 ± 1.99	3.11 ± 1.83	0.882	< 0.001
Orthogonal angulation	2.49 ± 1.96	2.74 ± 1.98	0.911	< 0.001
Classic 4-chamber angulation	3.26 ± 2.28	3.08 ± 2.36	0.910	< 0.001

Data are expressed as mean and standard deviation

*CC* correlation coefficient

*P values indicating the level of correlation

### Values of aortic distensibility among two observers

Figure 1S illustrates the distribution of AD values obtained by two observers depending on the angulation used at the time of image acquisition. Table 2 outlines the differences in absolute values of AD between two observers. In both observers, the classic 4-chamber AD differed from the strictly orthogonal cuts by about 25% when using traced aortic contours for AD calculation (3.26 ± 2.28 vs. 2.49 ± 1.96 10^−3^ mmHg^−1^ for observer 1 and 2.93 ± 2.37 vs. 2.35 ± 2.06 10^−3^ mmHg^−1^ for observer 2). As expected, this difference was smaller in the corresponding values for AD based on aortic diameter (about 12%). Pearson’s values for correlation between orthogonal AD and 4-chamber AD as well as for transversal AD and 4-chamber are listed in Table 3.

**Table 2:** Aortic area- and aortic diameter-derived measurements of aortic distensibility (AD) in two observers

Parameter (*n* = 31)	Observer 1	Observer 2	Pearson CC	*P* value CC*
AD (10^−3^ mmHg^−1^) based on aortic area				
Transversal angulation	2.80 ± 1.99	2.26 ± 2.06	0.895	< 0.001
Orthogonal angulation	2.49 ± 1.96	2.35 ± 2.06	0.921	< 0.001
Classic 4-chamber angulation	3.26 ± 2.28	2.93 ± 2.37	0.948	< 0.001
AD (10^−3^ mmHg^−1^) based on aortic diameter				
Transversal angulation	3.11 ± 1.84	2.61 ± 1.78	0.854	< 0.001
Orthogonal angulation	2.74 ± 1.99	2.62 ± 2.10	0.928	< 0.001
Classic 4-chamber angulation	3.08 ± 2.36	2.85 ± 2.29	0.949	< 0.001

Data are expressed as mean and standard deviation

*CC* correlation coefficient

*P values indicating the level of correlation

**Table 3:** Pearson’s values for correlation between orthogonal AD and classic 4-chamber AD as well as for transversal AD and classic 4-chamber AD

	Observer 1	Observer 2
	4-chamber angulation	4-chamber angulation
Area-based AD (10^−3^ mmHg^−1^)		
Orthogonal angulation	Pear = 0.92 (*R*^2^ = 0.8451)	Pear = 0.92 (*R*^2^ = 0.8451)
Transversal angulation	Pear = 0.90 (*R*^2^ = 0.8067)	Pear = 0.90 (*R*^2^ = 0.805)
Diameter-based AD (10^−3^ mmHg^−1^)		
Orthogonal angulation	Pear = 0.87 (*R*^2^ = 0.7558)	Pear = 0.90 (*R*^2^ = 0.8175)
Transversal angulation	Pear = 0.80 (*R*^2^ = 0.6412)	Pear = 0.85 (*R*^2^ = 0.7187)

*Pear* Pearson’s correlation coefficient

**Fig. 2:**
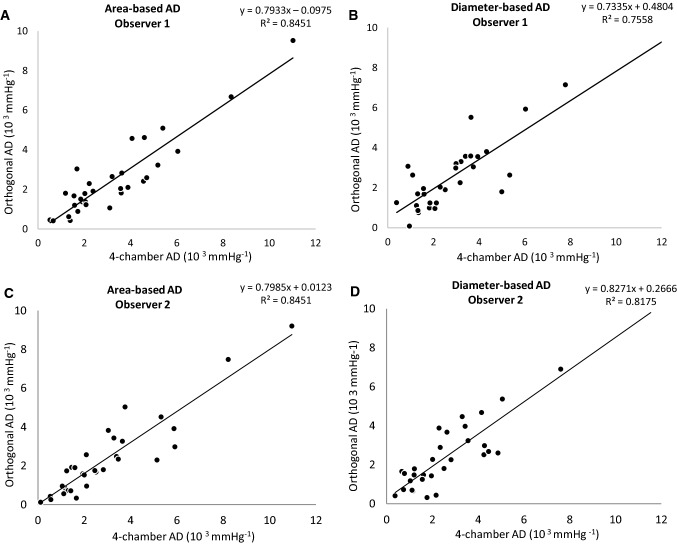
Correlation between orthogonal AD and the classic 4-chamber AD with the corresponding *R*^2^ values. Results are provided for Observer 1 (A + B) and Observer 2 (C + D) for both area-based AD and diameter-based AD

Interestingly, the highest values for correlation and reproducibility were found for 4-chamber AD measures both in area- and in diameter-based measurements. Figure 2 provides the correlation between orthogonal AD and the classic 4-chamber AD for both observers. For observer 1, the corresponding *R*^2^ values were 0.8451 for area-based AD and 0.7558 for diameter-based AD. For observer 2, the respective *R*^2^ values were 0.8451 and 0.8175.

### Intra- and inter-observer agreement

**Fig. 3:**
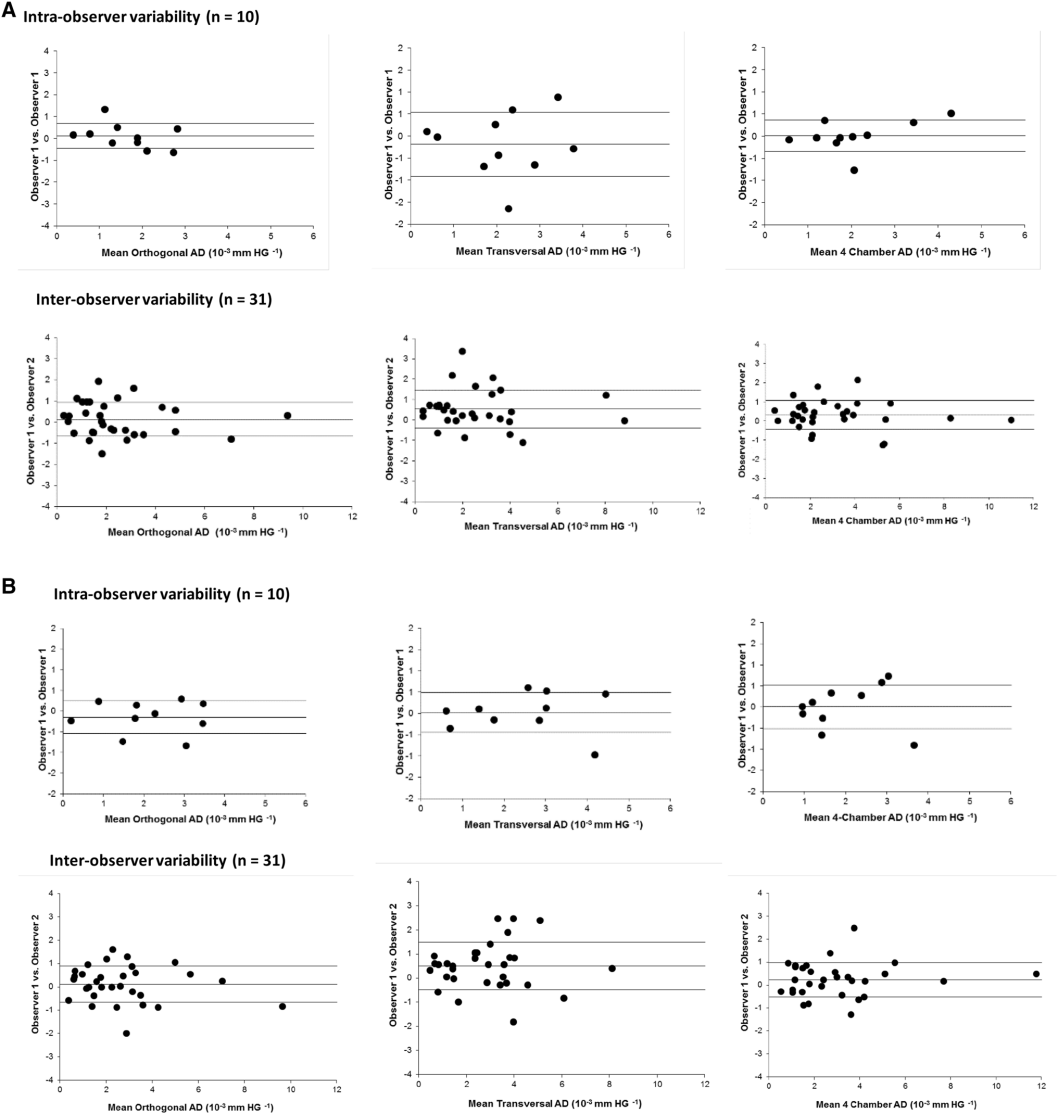
Bland–Altman plots demonstrating intra- and inter-observer variability for AD values obtained from contoured aortic areas (**a**) or diameter-based values (**b**) depending on the angulation of the aorta at the time of image acquisition

Figure 3 shows the Bland–Altman plots demonstrating intra- and inter-observer variability for AD values obtained from contoured (A) or diameter-based (B) aortic areas, depending on the angulation of the aorta at the time of image acquisition. Table 4 outlines the reproducibility giving the mean difference between two measurements and the corresponding intra-class correlation coefficient (ICC). Inter-observer agreement was excellent in both approaches used: for area-based AD, agreement was highest for 4-chamber AD (ICC 0.97; 95% CI 0.93–99), followed by orthogonal AD (ICC 0.96; 95% CI 0.91–98) and transversal AD (ICC 0.93; 95% CI 0.80–97). For diameter-based AD, agreement was also highest for 4-chamber AD (ICC 0.97; 95% CI 0.94–99), followed by orthogonal AD (ICC 0.96; 95% CI 0.92–98) and transversal AD (ICC 0.91; 95% CI 0.77–96).

**Table 4:** Intra-observer and inter-observer reproducibility for aortic distensibility based on aortic areas or diameters

Parameter	Mean difference ± SD	ICC (95% CI)
AD (10^−3^ mmHg^−1^) based on aortic area		
Intra-observer (*n* = 10)		
AD transversal angulation	− 0.19 ± 0.73	0.81 (0.41–0.95)
AD orthogonal angulation	0.11 ± 0.58	0.87 (0.50–0.97)
AD classic 4-chamber angulation	0.13 ± 0.35	0.97 (0.91–0.99)
Inter-observer (*n* = 31)		
AD transversal angulation	− 0.54 ± 0.94	0.93 (0.80–0.97)
AD orthogonal angulation	− 0.13 ± 0.81	0.96 (0.91–0.98)
AD classic 4-chamber angulation	− 0.32 ± 0.76	0.97 (0.93–0.99)
AD (10^−3^ mmHg^−1^) based on aortic diameter		
Intra-observer (*n* = 10)		
AD transversal angulation	0.02 ± 0.47	0.97 (0.89–0.99)
AD orthogonal angulation	− 0.14 ± 0.40	0.97 (0.88–0.99)
AD classic 4-chamber angulation	0.01 ± 0.52	0.93 (0.73–0.98)
Inter-observer (*n* = 31)		
AD transversal angulation	− 0.50 ± 0.98	0.91 (0.77–0.96)
AD orthogonal angulation	− 0.12 ± 0.78	0.96 (0.92–0.98)
AD classic 4-chamber angulation	− 0.23 ± 0.75	0.97 (0.94–0.99)

Data are expressed as mean and standard deviation. Mean difference = mean difference between the two measurements; coefficient of variability = SD of the mean difference between two measurements divided by the mean value of the parameter (Grothues et al. [19])

*ICC* intra-class correlation coefficient, CI = confidence interval

### Sample size calculation

Changes in reproducibility influence the sample size required to detect significant differences in AD. The sample sizes required for each AD value are given in Table 5.

**Table 5:** Sample size calculations for area-based AD and diameter-based AD to detect a clinically significant change of 0.5, 0.8 and one 10^−3^ mmHg^−1^ in aortic distensibility (with 90% power and an α error of 0.05)

Parameter	Mean difference ± SD	Sample size (*n*)		
		0.5	0.8	1
AD (10^−3^ mmHg^−1^) based on aortic area				
AD transversal angulation	− 0.54 ± 0.94	74	29	19
AD orthogonal angulation	− 0.13 ± 0.81	55	22	14
AD classic 4-chamber angulation	− 0.32 ± 0.76	48	19	12
AD (10^−3^ mmHg^−1^) based on the aortic diameter				
AD transversal angulation	− 0.50 ± 0.98	81	32	20
AD orthogonal angulation	− 0.12 ± 0.78	51	20	13
AD classic 4-chamber angulation	− 0.23 ± 0.75	47	18	12

Data are expressed as mean and standard deviation

*ICC* intra-class correlation coefficient, *CI* confidence interval, *SD* standard deviation

## Discussion

The present work was designed to investigate the reproducibility of AD calculation in standard 4-chamber cine CMR imaging. Our data demonstrate the following:

1. Assessment of AD through conventional 4-chamber cine images is easy and correlates highly with AD derived from a strictly orthogonal angulation of the aorta.
2. Excellent inter-observer and intra-observer reproducibility were observed for AD, irrespective of whether measurements were based on aortic diameter or manually traced aortic area.
3. The sample size calculation demonstrated a minimal number of *n* = 12 subjects to detect even small changes in AD.

Relationships of pulse pressure (PP) and flow are illustrated by impedance curves, which show higher frequencies when the aortic arch becomes stiffer and then reflects pulse waves earlier [21]. While determinants of peripheral resistance are simple to acquire, parameters of aortic stiffness require consideration of the distending PP, vascular tone and site of measurement [3]. As a gold standard for arterial stiffness, PWV requires certain geometric assumptions and extended planning and remains time consuming. Recently, 4D flow CMR has been shown to directly assess PWV in reduced time and to have a high correlation with AD values [22]. Measures of AD are relatively simple to obtain and reflect alterations in the central vasculature even in the absence of overt cardiovascular disease. No additional scans are needed to calculate AD. Thus, focusing on AD as a novel imaging biomarker for the prediction of cardiovascular risk has significant potential to improve individually adapted therapies [23].

While previous research aimed to assess AD in vivo, newer studies focus on therapeutic concepts to reduce arterial stiffness [11–14]. Both medical control of heart rate and modulation of the sympathetic nervous system by renal denervation have shown promising results to improve AD. [12–15] In preclinical studies, heart rate reduction with ivabradine has shown to improve arterial stiffness and diastolic function [14, 15]. Targeting AD might thus play an essential role in the management of heart failure with preserved ejection fraction (HFPEF).

**Fig. 4:**
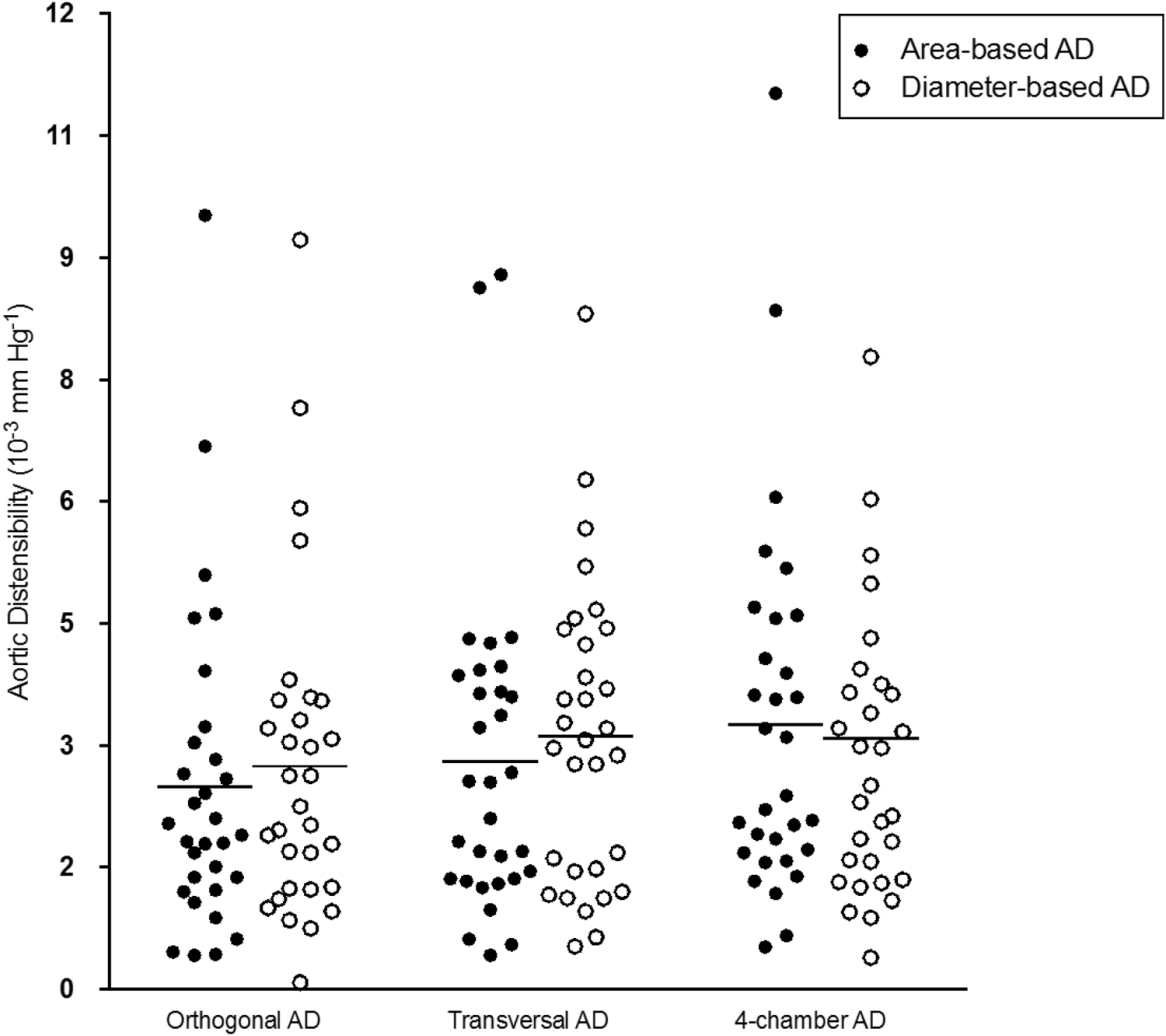
Distribution of AD values depending on the angulation used at the time of image acquisition. Aortic areas were acquired either by directly contoured aortic areas (black spots) or based on diameter measurements (white spots)

Reproducibility and accuracy of CMR were previously shown to be high, especially compared to those of echocardiography [24, 25]. We assumed that AD estimation would be feasible when focusing on images of the descending aorta that are available in every standard 4-chamber cine sequence of the heart (Fig. 1) [19]. For this purpose, we retrospectively analyzed already existing CMR data. Images were taken in diameter and area. Resulting values differed by about 15% (Fig. 4).

Based on the outlined data, we can assume that AD taken in standard 4-chamber cine images corresponds well with strictly orthogonal images but tends to overestimate values by about 25% (Table 3, Fig. 4). This deviation is a consequence of the geometry of cross-sectional vessel areas displayed with different angulations. While orthogonally derived areas are nearly circular, the areas in the 4-chamber view are based on an oblique section of the vessel in this projection. Values of inter- and intra-observer agreement were excellent in our data, underlining the high potential for retrospective analyses (Fig. 3). To give an impression of how this measurement technique will perform in daily practice, the correlation curves of two different observers are shown in Fig. 2.

CMR was shown to provide highly accurate and reproducible measures, especially when values are derived three times [18, 19, 26]. We used this strategy and averaged three measurements to improve reproducibility. Given the above evidence, AD obtained by tracing of the aortic wall is justified to be used in daily clinical practice for risk prediction.

Recently, two studies investigated AD measurement as a non-invasive tool to control the effect of renal denervation [4, 11]. These studies included 58 and 28 patients, respectively. Based on the present work, it seems that even fewer patients are necessary to detect small changes in AD when using conventional 4-chamber cine CMR images. The sample size calculation given in Table 5 shows that, e.g., 25 exams (*n* = 19 plus 25% dropout) per arm are necessary to detect a difference of 0.8 10^−3^ mmHg^−1^ in AD with a power of 90%. This makes our technique especially suitable for retrospective analyses of CMR datasets.

## Conclusions

AD measurements using conventional 4-chamber cine imaging are feasible and highly reproducible and reflect the elastic properties of the descending aorta accordingly. This allows an accurate and rapid assessment of arterial compliance and may help to predict changes in the central vasculature at an early stage, eventually preventing evolution toward left ventricular remodeling and dysfunction. Using automated contouring and 3D acquisition of the aorta, future trials could assess AD among different segments to increase accuracy and diagnostic value. In addition, our approach might be even used in a retrospective analysis of studies on already existing CMR data.

## Limitations

Among the major limits of our study is the relatively small number of patients included. A larger cohort could enhance the power of our results and outline more subtle differences among different angulations. We used a standard CMR cine sequence; the effect of different temporal and spatial resolutions of SSFP cine sequences on AD measurements has not been evaluated. There is a lack of comparison of our results with other markers of arterial compliance (e.g., PWV) and the absence of centrally measured BP. The invasiveness would, however, have limited the conduct of this study. Another limitation is the use of peripheral PP in our investigation. Central aortic PP would provide a more accurate absolute measure of AD.

## Electronic supplementary material

Below is the link to the electronic supplementary material.

Supplementary material 1 (PDF 278 kb)

## Data Availability

The datasets generated during and analyzed during the current study are available from the corresponding author on reasonable request.

## List of abbreviations

AA: Aortic areas
AC: Aortic compliance
AD: Aortic distensibility
BP: Blood pressure
CMR: Cardiac magnetic resonance tomography
ICC: Intra-class coefficient
MRI: Magnetic resonance imaging
PP: Pulse pressure
PWV: Pulse wave velocity
SD: Standard deviation
SSFP: Steady-state free precession sequence
