# Supplementary material for: Validation of simple measures of aortic distensibility based on standard 4-chamber cine CMR: a new approach for clinical studies
Source: Clin Res Cardiol. 2019 Jul 13;109(4):454–64. doi: 10.1007/s00392-019-01525-8 (PMC7098938; doi:10.1007/s00392-019-01525-8)

Figure 1S

Distribution of AD values obtained by two observers (black dots observer 1, white dots observer 2) depending on the angulation used at the time of image acquisition. Aortic areas were acquired either by directly contoured aortic areas (A) or based on diameter measurements (B).

Supplementary Figure

Figure 1S

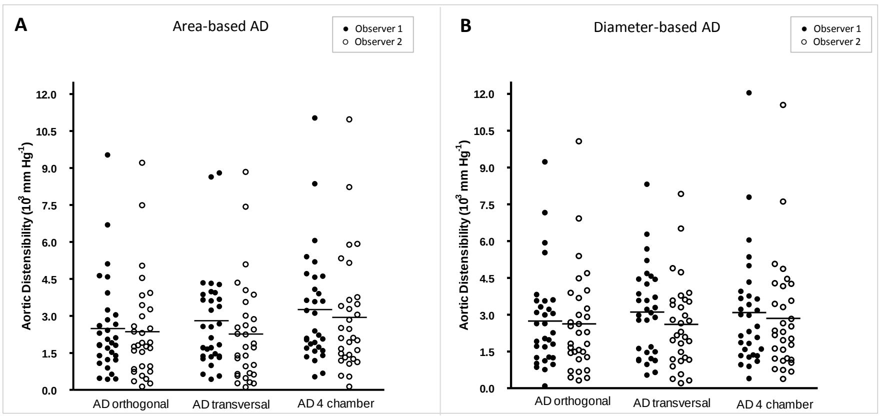

Supplement: Supplementary file 1 — Supplementary material 1 (PDF 278 kb) [file 392_2019_1525_MOESM1_ESM.pdf]
